# Association of *IGF1R* Gene Polymorphisms with Growth Traits in Yak

**DOI:** 10.3390/ani16203179

**Published:** 2026-10-10

**Authors:** Weiqiang Jiang, Yincang Han, Yonggang Sun, Xinyue Liu, Qingye Zhao, Shengwei Jin

**Affiliations:** 1 College of Agriculture and Animal Husbandry, Qinghai University, Xining 810016, China; 17325116086@163.com (W.J.); 18863739380@163.com (X.L.); 17662097713@163.com (Q.Z.); 2 Qinghai Provincial Key Laboratory of Conservation and Innovative Utilization of Livestock Genetic Resources in the Plateau, Xining 810016, China; sunyg410@163.com; 3 Academy of Animal Science and Veterinary Medicine, Qinghai University, Xining 810016, China; 4 Qinghai Province Livestock and Poultry Genetic Resources Protection and Utilization Center, Xining 810000, China; j13619704954@163.com

**Keywords:** yak, *IGF1R* gene, single-nucleotide polymorphism, growth traits, haplotype, diplotype

## Abstract

This study investigated polymorphisms in the insulin-like growth factor 1 receptor (*IGF1R*) gene and their associations with growth traits in yak. A total of 400 three-year-old female yaks were evaluated for body weight, withers height, body length, chest circumference, and cannon circumference. Four single-nucleotide polymorphisms (SNPs) were identified in the amplified region of exon 13 by PCR amplification followed by direct Sanger sequencing. Three loci exhibited moderate polymorphism and deviated from Hardy–Weinberg equilibrium, whereas g.7383182G>A showed low polymorphism and was in equilibrium. The CC genotype at g.7383089C>T was associated with significantly higher body weight, body length, and chest circumference than the CT and TT genotypes. The GA genotype at g.7383141G>A showed favorable values for several growth traits. At g.7383142T>C, the TT genotype showed higher body weight, body length, and chest circumference than the CC genotype. The GG genotype at g.7383182G>A was associated with greater withers height than the GA genotype. Seven common haplotypes were identified, with Hap4 being the most frequent (0.335). The H_4_H_1_ diplotype showed relatively favorable overall growth performance. These findings suggest that *IGF1R* variants may be candidate markers for yak growth traits, but validation in larger, independent populations is required.

## Simple Summary

Yaks are important livestock in the Qinghai–Tibetan Plateau, where they provide meat, milk, and other products under harsh environmental conditions. Growth performance is influenced by both environmental factors and genetic variations. In this study, we examined the *IGF1R* gene, which plays an important role in growth and development, in 400 three-year-old female yaks. Four genetic variants were identified and analyzed in relation to body weight and body measurements. Several variants were associated with differences in body weight, body length, withers height, chest circumference, or cannon circumference. We also identified seven common gene combinations and found that the H_4_H_1_ diplotype was associated with relatively favorable overall growth performance. These results provide preliminary information on the genetic factors that may influence yak growth and may assist the future development of molecular markers for genetic selection. However, the findings were obtained from a single farm population and should be validated in larger and independent yak populations before practical application.

## 1. Introduction

Yak (*Bos grunniens*) is an important livestock resource in the Qinghai–Tibetan Plateau and surrounding high-altitude regions. Yaks are well adapted to hypoxia, low temperatures, and extensive grazing conditions, and their meat, milk, and other products play important roles in the livestock industry of high-altitude areas. Genome-wide studies have revealed genetic features associated with high-altitude adaptation in yak [1], whereas whole-genome resequencing studies have provided important insights into yak domestication and population history [2]. Growth traits, including body weight, withers height, body length, and chest circumference, are complex quantitative traits jointly influenced by multiple genes and environmental factors. Genome-wide association studies have identified several genetic loci associated with growth traits in yak [3]. Genetic diversity and population structure differ among yak populations and may affect the distribution of alleles at candidate genes and their associations with phenotypic traits [4]. Genetic diversity in livestock populations is also influenced by demographic history, artificial and natural selection, genetic drift, and gene flow; therefore, the evaluation of candidate markers should take the genetic background of the target population into account [5]. In addition to conventional phenotypic measurement and genetic evaluation, the appropriate use of molecular markers and genomic information may improve the efficiency of livestock selection and genetic improvement [6].

The insulin-like growth factor system participates in cell proliferation, differentiation, metabolism, and tissue growth [7]. The *IGF1R* gene encodes a transmembrane receptor tyrosine kinase and represents an important component of this signaling system [8]. Binding of insulin-like growth factor ligands to *IGF1R* activates downstream signaling pathways, including the PI3K/AKT and MAPK pathways, which are involved in cell growth, survival, and metabolic regulation [9]. In humans, functional abnormalities in *IGF1R* have been associated with intrauterine and postnatal growth retardation, further demonstrating the importance of this gene in normal growth and development [10].

In livestock, genetic variations in *IGF1R* have been reported to be associated with various growth and production traits. Liang et al. [11] reported an association between *IGF1R* polymorphisms and growth traits in yak. Ma et al. [12] found that copy number variations in *IGF1R* were associated with body weight and body measurements in several Chinese beef cattle breeds. Luo et al. [13] reported associations between *IGF1R* polymorphisms and withers height, body length, and dairy-related traits in indigenous Chinese dairy goats. In addition, associations between *IGF1R* genetic variation and average daily gain in Egyptian buffalo, growth and carcass traits in sheep, growth traits in Hulun Buir sheep, and performance traits in pigs have also been reported [14,15,16,17]. These findings support the potential role of *IGF1R* as a candidate gene for growth and production traits in livestock.

However, studies on genetic variations in different regions of the yak *IGF1R* gene, its haplotype structure, and the associations between multi-locus combinations and growth traits remain limited. Compared with the previous yak-specific study by Liang et al. [11], which examined a single *IGF1R* polymorphism, the present study extends this work by (i) examining four variants in the amplified region of exon 13, (ii) evaluating genetic diversity and linkage disequilibrium, (iii) constructing haplotype and diplotype structures, and (iv) assessing associations between multi-locus combinations and multiple growth traits. Therefore, the present study investigated 400 three-year-old female yaks from a farm population in Qilian County, Qinghai Province, China. The amplified region of exon 13 of the *IGF1R* gene was screened for SNPs, and genetic diversity, linkage disequilibrium, and haplotype structure were analyzed. In addition, associations between different genotypes or diplotypes and growth traits were evaluated. The results provide basic information for the identification of candidate molecular markers associated with growth traits in yak.

## 2. Materials and Methods

### 2.1. Experimental Materials

A total of 400 healthy three-year-old female yaks were selected from a yak farm population in Yeniugou Township, Qilian County, Haibei Prefecture, Qinghai Province, China. All experimental animals came from the same farm population and were of the same age and sex, with good health status. During the experimental period, body weight, withers height, body length, chest circumference, and cannon circumference were measured for each animal.

Approximately 10 mL of blood was collected from the jugular vein of each experimental animal. Acid citrate dextrose was used as the anticoagulant, with a blood-to-anticoagulant volume ratio of 6:1. The samples were gently shaken, mixed, and stored at −80 °C until further use.

### 2.2. Genomic DNA Extraction and Detection

Genomic DNA was extracted using a blood DNA extraction kit (Tiangen Biotech Co., Ltd., Beijing, China). DNA integrity was examined by electrophoresis on a 1% agarose gel, and DNA concentration and purity were determined using a microvolume spectrophotometer. DNA samples that met the quality requirements were stored at −20 °C for subsequent PCR amplification.

### 2.3. Primer Design and PCR Amplification

Based on the yak *IGF1R* gene sequence in GenBank (NC_091637.1), PCR primers were designed using Primer 5.0 software (Table 1). The primers were synthesized by Sangon Biotech Co., Ltd. (Shanghai, China).

The 25.0-μL PCR reaction system contained 12.5 μL of PCR Mix, including dNTPs, Taq DNA polymerase, and 10× Buffer, 0.7 μL of each primer at 10 pmol/μL, 1.0 μL of template DNA at 50 ng/μL, and 10.1 μL of ddH_2_O.

The PCR reaction conditions were as follows: pre-denaturation at 94 °C for 4 min; denaturation at 94 °C for 30 s, annealing at 58.7 °C for 30 s, and extension at 72 °C for 30 s for 35 cycles; final extension at 72 °C for 10 min; and storage at 4 °C. The PCR amplification products were detected by electrophoresis on a 0.8% agarose gel.

### 2.4. SNP Detection

PCR products with good amplification quality were purified and submitted to Sangon Biotech Co., Ltd. (Shanghai, China) for direct Sanger sequencing. The sequencing results were compared and analyzed using SeqMan Pro software.(version 11.1.0) Candidate nucleotide substitutions were identified by comparison with the reference yak *IGF1R* sequence NC_091637.1. Homozygous genotypes were identified by a single clear peak at the polymorphic position, whereas heterozygous genotypes were identified by two overlapping peaks.

### 2.5. Statistical Analysis

Genotype frequencies, allele frequencies, expected heterozygosity (He), effective number of alleles (Ne), and polymorphism information content (PIC) were calculated based on the genotype data. PIC was used to evaluate the ability of genetic markers to reflect genetic diversity in the population [18]. Linkage disequilibrium and haplotype analyses were performed using Haploview 3.32 software [19]. D′ and r^2^ were used to describe the linkage disequilibrium between loci. Haplotypes with frequencies below 3.00% were excluded from the analysis, and diplotypes meeting the inclusion criterion were analyzed for their associations with growth traits.

The distribution of *IGF1R* genotypes in the yak population was analyzed using the chi-square test in SPSS 25.0. When χ^2^ < χ^2^_0.05_, the locus was considered to be in Hardy–Weinberg equilibrium. When χ^2^_0.05_ ≤ χ^2^ < χ^2^_0.01_, the locus was considered to be in Hardy–Weinberg disequilibrium. When χ^2^ ≥ χ^2^_0.01_, the locus was considered to be in highly significant Hardy–Weinberg disequilibrium.

Associations between *IGF1R* genotypes and growth traits were evaluated using one-way analysis of variance (ANOVA), with genotype treated as a fixed effect. When the overall genotype effect was significant, post hoc multiple comparisons were performed. Because all animals were three-year-old females from the same farm population, age, sex, and farm were constant and were not included as independent explanatory variables in the association model. The revised statistical model was:

Y_ij_ = μ + G_i_ + E_ij_

where Y_ij_ is the phenotypic record of an individual; μ is the population mean; G_i_ is the marker genotype effect; and E_ij_ is the random error.

To account for multiple testing, the Benjamini–Hochberg false discovery rate (BH-FDR) procedure was applied to the 20 genotype–trait association tests involving four SNP loci and five growth traits. The five diplotype–trait association tests were treated as a separate multiple-testing family and corrected independently. An FDR-adjusted *p* value < 0.05 was considered statistically significant.

## 3. Results

### 3.1. Identification of SNP Loci in the Yak IGF1R Gene

**Figure 1:**
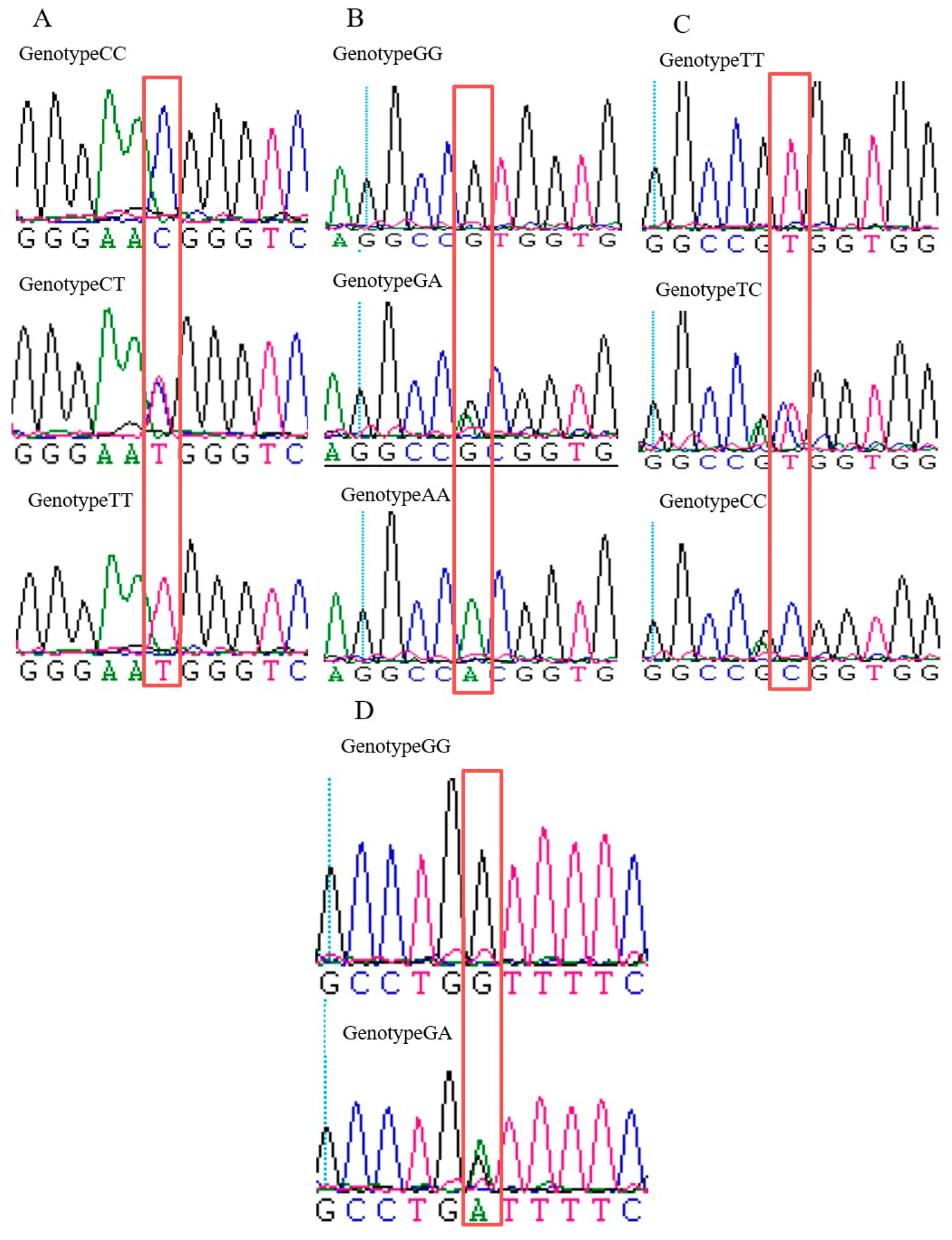
Sequencing chromatograms of the four loci in the yak *IGF1R* gene: (**A**) g.7383089C>T; (**B**) g.7383141G>A; (**C**) g.7383142T>C; (**D**) g.7383182G>A.

The yak *IGF1R* gene is 300,676 bp in length and consists of 21 exons. Sequence analysis identified four SNP loci in exon 13 of the yak *IGF1R* gene (Figure 1), namely g.7383089C>T, g.7383141G>A, g.7383142T>C, and g.7383182G>A.

Three genotypes, CC, CT, and TT, were identified at g.7383089C>T. Three genotypes, GG, GA, and AA, were identified at g.7383141G>A. Three genotypes, TT, TC, and CC, were identified at g.7383142T>C. Two genotypes, GG and GA, were identified at g.7383182G>A.

### 3.2. Genetic Polymorphism of the IGF1R SNP Loci

At the g.7383089C>T locus, the frequencies of the CC, CT, and TT genotypes were 66.75%, 25.00%, and 8.25%, respectively. The frequencies of the C and T alleles were 79.25% and 20.75%, respectively. The He, Ne, and PIC values were 0.3289, 1.4901, and 0.2748, respectively, indicating moderate polymorphism.

At the g.7383141G>A locus, the frequencies of the GG, GA, and AA genotypes were 50.00%, 33.25%, and 16.75%, respectively. The frequencies of the G and A alleles were 66.63% and 33.38%, respectively. The He, Ne, and PIC values were 0.4447, 1.8009, and 0.3458, respectively, indicating moderate polymorphism.

At the g.7383142T>C locus, the frequencies of the TT, TC, and CC genotypes were 50.00%, 25.00%, and 25.00%, respectively. The frequencies of the T and C alleles were 62.50% and 37.50%, respectively. The He, Ne, and PIC values were 0.4688, 1.8824, and 0.3589, respectively, indicating moderate polymorphism.

At the g.7383182G>A locus, the frequencies of the GG and GA genotypes were 91.75% and 8.25%, respectively. The frequencies of the G and A alleles were 95.88% and 4.13%, respectively. The He, Ne, and PIC values were 0.0791, 1.0859, and 0.0760, respectively, indicating low polymorphism.

The results of the Hardy–Weinberg equilibrium test showed that the g.7383182G>A locus, with a χ^2^ value of 0.7405, did not significantly deviate from Hardy–Weinberg equilibrium. The χ^2^ values of g.7383089C>T, g.7383141G>A, and g.7383142T>C were 23.0134, 25.4705, and 87.1111, respectively, and all three loci significantly deviated from Hardy–Weinberg equilibrium (Table 2). Because all animals were sampled from the same farm population, these deviations cannot be explained by differences among sampling locations. Possible explanations include non-random mating, cryptic relatedness, inbreeding, genetic drift, genotyping error, or locus-specific selection; however, the present study design does not allow these explanations to be distinguished.

### 3.3. Effects of IGF1R Genotypes on Growth Traits in Yak

As shown in Table 3, at the g.7383089C>T locus, the body weight, body length, and chest circumference of individuals with the CC genotype were significantly higher than those of individuals with the CT and TT genotypes (*p* < 0.01). The withers height of individuals with the CC genotype was significantly higher than that of individuals with the CT genotype (*p* < 0.05), and their cannon circumference was significantly higher than that of individuals with the CT and TT genotypes (*p* < 0.05).

At the g.7383141G>A locus, the body weight, body length, and chest circumference of individuals with the GA genotype were highly significantly greater than those of individuals with the GG genotype (*p* < 0.01). The withers height of individuals with the GA genotype was highly significantly greater than that of individuals with the GG and AA genotypes (*p* < 0.01), and their cannon circumference was significantly greater than that of individuals with the GG genotype (*p* < 0.05). The body weight, withers height, body length, and chest circumference of individuals with the AA genotype were highly significantly lower than those of individuals with the GA genotype (*p* < 0.01).

At the g.7383142T>C locus, the body weight and chest circumference of individuals with the TT genotype were highly significantly greater than those of individuals with the CC genotype (*p* < 0.01), and their body length was significantly greater than that of individuals with the CC genotype (*p* < 0.05). The body weight and cannon circumference of individuals with the TC genotype were significantly greater than those of individuals with the CC genotype (*p* < 0.05). No significant differences were observed for the other traits (*p* > 0.05).

At the g.7383182G>A locus, the withers height of individuals with the GG genotype was highly significantly greater than that of individuals with the GA genotype (*p* < 0.01). The body weight, body length, chest circumference, and cannon circumference of individuals with the GG genotype were also greater than those of individuals with the GA genotype, but the differences did not reach statistical significance (*p* > 0.05).

To account for multiple testing, the Benjamini–Hochberg FDR procedure was applied to the 20 genotype–trait association tests. After FDR correction, 18 of the 20 associations remained significant (adjusted *p* < 0.05). The associations between g.7383142T>C and withers height (adjusted *p* = 0.4077) and between g.7383142T>C and cannon circumference (adjusted *p* = 0.07105) were not significant after correction; these are therefore described as suggestive associations. All other genotype–trait associations remained significant after FDR adjustment, including the associations of g.7383089C>T (CC genotype) and g.7383141G>A (GA genotype) with body weight, body length, and chest circumference.

### 3.4. Haplotype and Linkage Disequilibrium Analysis of the IGF1R SNP Loci

Haplotype analysis was performed for the four SNP loci in the yak *IGF1R* gene. Haplotypes with frequencies below 3.00% were excluded. Seven haplotypes, designated Hap1–Hap7, were identified (Table 4). Hap4 had the highest frequency, at 0.335, and was the major haplotype. Hap5 had the lowest frequency, at 0.034. The seven haplotypes shown accounted for a combined frequency of 0.933; the remaining frequency of 0.067 corresponded to rare haplotypes that were not displayed individually.

Linkage disequilibrium analysis showed that there was no strong linkage among the four SNP loci (Table 5). The pairwise D′ values among the four SNP loci were below 0.8, and the r^2^ values were below 0.33. Strong linkage disequilibrium was considered to occur when D′ > 0.8 and r^2^ > 0.33.

### 3.5. Effects of Combined Haplotypes of the IGF1R SNP Loci on Yak Growth Traits

A total of 19 combined haplotypes were identified based on the four SNP loci in the yak *IGF1R* gene. Thirteen combined haplotypes with frequencies below 3.00% were defined as other types and excluded. The remaining combined haplotypes were analyzed for their associations with yak growth traits.

As shown in Table 6, the mean values of body weight, chest circumference, and cannon circumference for individuals with the combined haplotypes H_4_H_1_, H_4_H_2_, H_4_H_3_, and H_4_H_4_ were significantly higher than those for individuals with the H3H3 and H_4_H_7_ combined haplotypes (*p* < 0.05). The mean values of withers height and body length for individuals with the H_4_H_1_ and H_4_H_2_ combined haplotypes were significantly higher than those for individuals with the H_3_H_3_, H_4_H_3_, H_4_H_4_, and H_4_H_7_ combined haplotypes (*p* < 0.05). H_4_H_1_ was the diplotype with the best overall performance, showing the highest values for body weight, withers height, chest circumference, and cannon circumference.

## 4. Discussion

### 4.1. Genetic Polymorphism of the Yak IGF1R Gene

Four SNP loci were identified in the amplified region of exon 13 of the yak *IGF1R* gene, of which three loci exhibited moderate polymorphism, and g.7383182G>A exhibited low polymorphism. Chai et al. [20] performed whole-genome resequencing of indigenous domestic yaks on the Qinghai–Tibetan Plateau and analyzed the evolution and genetic differentiation of different populations, revealing different genetic backgrounds among local yak populations. Compared with the whole-genome analysis in that study, the present study only examined a local candidate-gene region in a single farm population and therefore cannot compare allele-frequency differences among different populations.

The first three loci significantly deviated from Hardy–Weinberg equilibrium, and the observed heterozygote frequencies were lower than the expected frequencies calculated from the allele frequencies. Although all animals were sampled from the same farm and were matched for age and sex, pedigree information, family structure information, and genome-wide genotype data were unavailable. Therefore, population stratification, cryptic relatedness, non-random mating, and inbreeding could not be formally controlled for in the present analysis. The causes of the deviations from Hardy–Weinberg equilibrium cannot be determined. These deviations may be related to population structure, sampling bias, genetic drift, or selection, and further validation in multiple independent populations is required. Consequently, the observed genotype–trait associations may partly reflect family structure or local genetic background and should not be interpreted as definitive causal effects.

Because the present study used a single-farm candidate-gene design, the results may be affected by unmeasured relatedness or population structure. Future studies should incorporate larger and independent populations and use mixed-effect models, pedigree information, or genomic relationship matrices to reduce this potential bias.

### 4.2. Associations Between IGF1R SNPs and Yak Growth Traits

After Benjamini–Hochberg FDR correction, 18 of the 20 genotype–trait associations remained significant. The associations between g.7383142T>C and withers height and between g.7383142T>C and cannon circumference were no longer significant after correction and are therefore described as suggestive associations. All other genotype–trait associations, including the key associations of g.7383089C>T (CC genotype) and g.7383141G>A (GA genotype) with body weight, body length, and chest circumference, remained significant after FDR adjustment.

In the present study, individuals with the CC genotype at g.7383089C>T showed relatively favorable performance in body weight, body length, and chest circumference, suggesting that this locus or the local genetic background represented by it may be associated with individual growth. Using gene-disruption models in mice, Baker et al. [21] found that *IGF* ligands and their receptors play important roles in embryonic and postnatal growth, and that functional deficiencies in related genes can cause significant growth defects. These findings provide functional evidence supporting the importance of the *IGF* system in normal growth and are biologically consistent with the association between *IGF1R* genetic variations and growth traits observed in the present study. However, gene disruption and SNPs occurring naturally in a population are not the same type of genetic change. The former cannot directly demonstrate that the C allele in the present study enhances receptor function. The differences observed in this study may be caused by the locus itself or by linkage between this locus and an unexamined functional variant.

The traits associated with the different SNPs in the present study were not completely consistent. In particular, the g.7383182G>A locus showed a clear difference in withers height. Using mice with combined deficiencies in the growth hormone receptor, *IGF-1*, and related pathways, Lupu et al. [22] found that growth hormone and *IGF-1* play both interrelated and non-redundant roles in postnatal growth, and that their contributions vary with growth stage. In comparison with that study, the present study measured body weight and body measurements at a single time point in three-year-old female yaks and therefore cannot determine when the genotype-associated differences were established. Differences in the association patterns among traits may be related to the different developmental schedules of bone, muscle, and other tissues, and may also be affected by sample size and phenotypic measurement error. Therefore, longitudinal growth records are needed to determine whether these loci have stage-specific effects.

Regarding muscle metabolism, Stitt et al. [23] found that the *IGF-1*/*PI3K/AKT* pathway inhibits *FOXO* transcription factors and reduces the expression of ubiquitin ligases associated with muscle atrophy, indicating that *IGF* signaling affects not only tissue synthesis but also the regulation of muscle protein degradation. The relatively favorable body weight and chest circumference observed in some genotypes in the present study are consistent with the functional background of the *IGF* system in tissue growth.

At the g.7383141G>A locus, the GA genotype showed relatively favorable values for several growth traits, indicating that the heterozygous genotype performed better. However, this observation alone cannot be directly interpreted as an overdominant effect. Le Roith et al. [24] pointed out in their comprehensive analysis of the growth hormone–IGF system that individual growth is not determined solely by circulating *IGF-1*, but also involves locally produced *IGF*, paracrine and autocrine effects, and endocrine feedback. Based on this understanding, genotype-associated differences in the present study may be affected by tissue-specific responses and physiological status, rather than following a simple allele-dose relationship. At the same time, the higher mean values observed for the GA genotype may also be related to linkage background and uncontrolled family differences. Further evaluation using genetic models and independent population validation is required to determine the mode of action.

The TT genotype at g.7383142T>C showed higher mean values for body weight, body length, and chest circumference than the CC genotype, indicating that this locus may be associated with several correlated growth indicators. However, body weight, body length, and chest circumference are phenotypically correlated traits. Therefore, the association of one locus with multiple traits does not necessarily indicate that it independently controls several biological processes. Multi-trait analysis and body-composition measurements could be used in future studies to distinguish an overall body-size effect from an effect on a specific tissue.

It should be emphasized that the present study did not determine whether the identified variants are located in a specific fibronectin type III (FnIII) domain of *IGF1R*, nor did it measure *IGF1R* expression, receptor phosphorylation, ligand-binding affinity, or PI3K/AKT activity. Although *IGF1R* is a transmembrane receptor tyrosine kinase whose extracellular region contains several structural and ligand-interacting regions, including FnIII domains, and whose intracellular kinase domain mediates downstream signal transduction, the association results obtained in this study cannot demonstrate that any specific SNP directly alters receptor signaling. For a missense variant, a potential amino-acid substitution could theoretically affect local protein structure, residue conservation, receptor folding, protein stability, or domain–domain interactions; for a synonymous variant, the amino-acid sequence would remain unchanged, but mRNA splicing, transcript stability, codon usage, translation efficiency, or regulatory motifs could potentially be affected. These possible effects were not tested in the present study and require validation using sequence conservation analysis, protein-structure prediction, splicing prediction, gene-expression analysis, reporter assays, receptor-binding assays, or cellular phosphorylation assays.

### 4.3. Significance of Haplotype and Diplotype Combination Analysis

Seven common haplotypes were retained in the present study, among which Hap4 had the highest frequency. Diplotype analysis showed that individuals with H_4_H_1_ had relatively high mean values for body weight, withers height, chest circumference, and cannon circumference, whereas individuals with H_4_H_2_ had the highest mean value for body length. Sutter et al. [25] reported, through genetic mapping in dogs, that a haplotype in the *IGF1* region was strongly associated with small body size. A common feature of that study and the present study is that multi-locus genetic combinations may carry information related to body size. However, the canine study involved *IGF1* rather than *IGF1R*, and dog breeds have undergone strong artificial selection for body size. Therefore, the effects of the haplotype reported in dogs cannot be directly extrapolated to yak. The differences among diplotypes in the present study may reflect local genetic background or linkage with unexamined functional variants, but they do not yet demonstrate a synergistic effect among the four SNPs.

Gray et al. [26] further compared genetic variations in the *IGF1* region between dogs and wolves and investigated the relationship between the small-dog haplotype and the genetic background of Middle Eastern grey wolves. Their findings showed that the distribution of body-size-related haplotypes is also affected by population origin and historical processes. In comparison, the present study did not analyze the origin of haplotypes across populations. Therefore, the high frequency of Hap4 cannot be directly interpreted as evidence that it was favored by selection for superior growth. Its distribution may also be affected by founder effects, genetic drift, or gene flow. In addition, all common haplotypes listed in Table 4 carried the G allele at g.7383182G>A. Therefore, the diplotype comparisons shown in Table 6 cannot directly evaluate the combined effect of the A allele at this locus.

The D′ and r^2^ values among the four loci in the present study were generally low. Qanbari [27] reported in a review of linkage disequilibrium in livestock that linkage disequilibrium is affected by population history, effective population size, selection, recombination, and allele frequencies. Compared with livestock populations that have undergone strong selection and possess relatively long linkage disequilibrium regions, the region examined in the present study showed relatively weak linkage disequilibrium.

### 4.4. Limitations of the Present Study

The present study has several limitations. First, all four variants were genotyped using direct Sanger sequencing, which is appropriate for variant discovery and confirmation but is less cost-effective and less scalable than allele-specific or probe-based methods for large-scale screening. Future studies should use high-throughput genotyping methods, such as Kompetitive Allele-Specific PCR (KASP), TaqMan allelic discrimination, PCR-RFLP, or high-resolution melting analysis, for routine screening of these loci. Among these approaches, KASP is a promising option for large-scale validation of the four known biallelic variants. Second, the study population was collected from a single farm, and no independent validation population was included. Third, the functional effects of the variants were not experimentally examined. Fourth, population stratification, cryptic relatedness, and inbreeding could not be formally controlled because genome-wide marker data and complete pedigree information were unavailable. Therefore, the present findings should be regarded as preliminary and hypothesis-generating rather than definitive evidence of causal effects. Further validation using larger and independent yak populations, pedigree information, genome-wide markers, and functional assays is required before these variants can be applied in molecular breeding or yak breed-resource evaluation.

## 5. Conclusions

Four SNP loci were successfully identified in exon 13 of the yak *IGF1R* gene. The loci g.7383089C>T, g.7383141G>A, and g.7383142T>C exhibited moderate polymorphism, whereas g.7383182G>A exhibited low polymorphism. After Benjamini–Hochberg FDR correction, 18 of the 20 genotype–trait associations remained significant. The associations between g.7383142T>C and withers height or cannon circumference were not significant after correction. The CC genotype at g.7383089C>T, the GA genotype at g.7383141G>A, and the TT genotype at g.7383142T>C showed relatively favorable performance for several growth traits. Diplotype analysis based on the four SNP loci showed that associations with body weight, withers height, body length, and chest circumference remained significant after FDR correction, whereas the association with cannon circumference did not. Individuals with the H_4_H_1_ diplotype had relatively favorable overall growth performance and may represent a potential favorable genetic combination associated with yak growth traits. In conclusion, the four SNP loci in the yak *IGF1R* gene and their diplotype combinations have potential as candidate molecular markers for growth traits.

## Figures and Tables

**Table 1:** Sequence information of primers.

Primer	Primer Sequence (5′–3′)	Fragment Length/bp	Annealing Temperature/°C	Amplification Region
*IGF1R*	F:AGTACGGAGGAGCCAAGCTA	207	58.7	Exon 13
	R:CTCTTCTCGGTGGTGGGTGAT			

**Table 2:** Genetic polymorphism analysis of the *IGF1R* gene in yak.

Loci	Genotypic Frequencies (%)			Allelic Frequencies (%)		χ^2^	(PIC)	He	Ne
g.7383089C>T	CC (267)	CT (100)	TT (33)	C	T	23.0134	0.2748	0.3289	1.4901
	66.75	25	8.25	79.25	20.75				
g.7383141G>A	GG (200)	GA (133)	AA (67)	G	A	25.4705	0.3458	0.4447	1.8009
	50	33.25	16.75	66.63	33.38				
g.7383142T>C	TT (200)	TC (100)	CC (100)	T	C	87.1111	0.3589	0.4688	1.8824
	50	25	25	62.5	37.5				
g.7383182G>A	GG (367)	GA (33)		G	A	0.7405	0.0760	0.0791	1.0859

PIC > 0.5 indicates high diversity; 0.25 < PIC < 0.5 indicates moderate diversity; and PIC < 0.25 indicates low diversity. χ^2^_0.05_ = 5.991; χ^2^_0.01_ = 9.21.

**Table 3:** Effect of different genotypes of *IGF1R* on growth traits in yak.

Loci	Gene Type	Body Weight/kg	Withers Height/cm	Body Length/cm	Chest Circumference/cm	Circumference/cm
g.7383089C>T	CC (*n* = 267)	140.41 ± 16.81 A	99.14 ± 5.77 Aa	105.76 ± 9.67 A	138.10 ± 12.06 A	15.05 ± 0.85 a
	CT (*n* = 100)	128.07 ± 16.56 B	96.20 ± 5.31 B	100.33 ± 7.06 B	127.81 ± 14.27 B	14.84 ± 0.79 b
	TT *(n* = 33)	127.51 ± 13.74 B	96.83 ± 5.33 b	100.91 ± 6.48 B	130.38 ± 12.95 B	14.67 ± 0.58 b
g.7383141G>A	GG (*n* = 200)	135.32 ± 16.98 B	97.96 ± 5.64 Ba	103.22 ± 7.60 B	134.16 ± 13.82 Ba	14.87 ± 0.80 A
	GA (*n* = 133)	141.74 ± 17.48 A	99.68 ± 6.10 A	106.75 ± 11.40 A	138.35 ± 11.86 A	15.12 ± 0.93 B
	AA (*n* = 67)	128.20 ± 15.58 C	96.07 ± 4.59 Bb	100.90 ± 6.99 B	130.19 ± 13.97 Bb	14.90 ± 0.59
g.7383142T>C	TT (*n* = 200)	137.78 ± 17.34 A	98.55 ± 5.65	104.88 ± 9.92 A	136.34 ± 12.81 A	14.97 ± 0.83
	TC (*n* = 100)	137.39 ± 15.76 a	98.18 ± 5.86	104.57 ± 7.98 a	136.14 ± 12.38 AB	15.09 ± 0.82 a
	CC (*n* = 100)	132.10 ± 18.93 Bb	97.60 ± 5.90	101.67 ± 8.44 Bb	130.74 ± 15.09 C	14.82 ± 0.81 b
g.7383182G>A	GG (*n* = 367)	137.58 ± 17.16	98.61 ± 5.78 A	104.57 ± 9.24	135.99 ± 12.91	14.99 ± 0.84
	GA (*n* = 33)	121.55 ± 14.39	93.86 ± 3.25 B	97.73 ± 5.54	122.61 ± 13.98	14.64 ± 0.52

Values are presented as means ± standard deviation. Different lowercase superscript letters within the same column indicate significant differences (*p* < 0.05), whereas different uppercase superscript letters indicate highly significant differences (*p* < 0.01), based on post hoc multiple comparisons. The overall ANOVA *p* values and Benjamini–Hochberg FDR-adjusted *p* values for the genotype–trait associations are provided in Appendix A Table A1. Associations with an FDR-adjusted *p* value < 0.05 were considered statistically significant. After FDR correction, the associations between g.7383142T>C and withers height or cannon circumference were not significant; therefore, no superscript letters are shown for these comparisons. Genotype groups with small sample sizes, particularly g.7383089C>T-TT (*n* = 33) and g.7383182G>A-GA (*n* = 33), may have reduced statistical power and less precise estimates; results involving these rare genotypes should therefore be interpreted cautiously.

**Table 4:** Haplotype Analysis of *IGF1R* Gene Mutation Site in yak.

Haplotype	g.7383089C>T	g.7383141G>A	g.7383142T>C	g.7383182G>A	Frequency
Hap1	C	A	C	G	0.099
Hap2	C	A	T	G	0.156
Hap3	C	G	C	G	0.179
Hap4	C	G	T	G	0.335
Hap5	T	A	T	G	0.034
Hap6	T	G	C	G	0.05
Hap7	T	G	T	G	0.08

Haplotypes with frequencies below 3.00% were excluded from the table. The seven haplotypes shown accounted for a combined frequency of 0.933; the remaining frequency of 0.067 corresponded to rare haplotypes that were not displayed individually.

**Table 5:** Analysis of *IGF1R* mutation site linkage disequilibrium in yak.

Loci	g.7383141G>A		g.7383142T>C		g.7383182G>A	
	D′	r^2^	D’	r^2^	D′	r^2^
**g.7383089C>T**	0.007	0.001	0.101	0.004	0.301	0.015
**g.7383141G>A**	-	-	0.052	0.002	0.222	0.004
**g.7383142T>C**	-	-	-	-	0.211	0.003

**Table 6:** Correlation analysis of *IGF1R* mutation site diplotypes and growth traits in yak.

	Body Weight/kg	Withers Height/cm	Body Length/cm	Chest Circumference/cm	Circumference/cm
H_3_H_3_ (30)	137.91 ± 16.17 ^Ba^	99.6 ± 6.12 ^a^	104.57 ± 8.22 ^b^	134.68 ± 12.36 ^a^	14.90 ± 0.78 ^b^
H_4_H_1_ (23)	146.33 ± 14.97 ^A^	101.04 ± 6.35 ^A^	108.65 ± 9.05 ^A^	140.52 ± 8.72 ^A^	15.28 ± 0.94 ^a^
H_4_H_2_ (50)	144.25 ± 17.27 ^A^	99.86 ± 6.30 ^A^	109.44 ± 14.85 ^Aa^	140.10 ± 11.49 ^A^	15.18 ± 0.89 ^a^
H_4_H_3_ (31)	141.50 ± 14.09 ^A^	98.68 ± 5.38 ^a^	105.39 ± 7.94 ^b^	140.00 ± 10.58 ^A^	15.15 ± 0.96 ^a^
H_4_H_4_ (64)	140.26 ± 17.31 ^A^	99.79 ± 5.50 ^A^	105.72 ± 7.30 ^b^	138.46 ± 12.64 ^A^	15.06 ± 0.80 ^a^
H_4_H_7_ (23)	128.27 ± 18.68 ^Bb^	95.41 ± 5.42 ^Bb^	99.91 ± 7.63 ^Bc^	127.41 ± 15.66 ^Bb^	14.63 ± 0.77 ^b^

Values are presented as means ± standard deviation. Different lowercase superscript letters within the same column indicate significant differences (*p* < 0.05), whereas different uppercase superscript letters indicate highly significant differences (*p* < 0.01), based on post hoc comparisons. The overall ANOVA *p* values and Benjamini–Hochberg FDR-adjusted *p* values for the diplotype–trait associations are provided in Appendix A Table A2. After FDR correction, the associations with body weight, withers height, body length, and chest circumference remained significant, whereas the association with cannon circumference was not significant.

## Data Availability

The raw data supporting the conclusions of this article will be made available by the authors on request.

## Abbreviations

The following abbreviations are used in this manuscript:

DNA	deoxyribonucleic acid
ddH_2_O	double-distilled water
He	expected heterozygosity
*IGF*	insulin-like growth factor
*IGF1*	insulin-like growth factor 1
*IGF1R*	insulin-like growth factor 1 receptor
Ne	effective number of alleles
PCR	polymerase chain reaction
PIC	polymorphism information content
*PI3K*	phosphoinositide 3-kinase
SNP	single-nucleotide polymorphism

## Appendix A

**Table A1:** Benjamini–Hochberg FDR-adjusted *p* values for associations between *IGF1R* genotypes and growth traits in yak.

Locus	Trait	Raw *p* Value	BH-Adjusted *p* Value	Significance
g.7383089C>T	Body weight	4.398 × 10^−11^	4.398 × 10^−10^	Significant
g.7383089C>T	Withers height	2.044 × 10^−5^	4.773 × 10^−5^	Significant
g.7383089C>T	Body length	2.306 × 10^−7^	1.153 × 10^−6^	Significant
g.7383089C>T	Chest circumference	2.275 × 10^−11^	4.398 × 10^−10^	Significant
g.7383089C>T	Cannon circumference	0.00916	0.01309	Significant
g.7383141G>A	Body weight	5.847 × 10^−7^	1.949 × 10^−6^	Significant
g.7383141G>A	Withers height	8.767 × 10^−5^	1.594 × 10^−4^	Significant
g.7383141G>A	Body length	2.148 × 10^−5^	4.773 × 10^−5^	Significant
g.7383141G>A	Chest circumference	0.000140	2.333 × 10^−4^	Significant
g.7383141G>A	Cannon circumference	0.0198	0.02329	Significant
g.7383142T>C	Body weight	0.0223	0.02478	Significant
g.7383142T>C	Withers height	0.4077	0.4077	Not significant
g.7383142T>C	Body length	0.01276	0.01701	Significant
g.7383142T>C	Chest circumference	0.00168	0.002585	Significant
g.7383142T>C	Cannon circumference	0.0675	0.07105	Not significant
g.7383182G>A	Body weight	3.125 × 10^−7^	1.250 × 10^−6^	Significant
g.7383182G>A	Withers height	4.629 × 10^−6^	1.323 × 10^−5^	Significant
g.7383182G>A	Body length	3.567 × 10^−5^	7.134 × 10^−5^	Significant
g.7383182G>A	Chest circumference	2.885 × 10^−8^	1.923 × 10^−7^	Significant
g.7383182G>A	Cannon circumference	0.0179	0.02238	Significant

Note: Raw *p* values were obtained from the overall ANOVA tests comparing growth traits among genotypes at each *IGF1R* locus. The Benjamini–Hochberg procedure was applied to the 20 genotype–trait tests. An FDR-adjusted *p* value < 0.05 was considered statistically significant. Eighteen of the 20 associations remained significant after FDR correction.

**Table A2:** Benjamini–Hochberg FDR-adjusted *p* values for associations between haplotype combinations and growth traits in yak.

Trait	Raw *p* Value	BH-Adjusted *p* Value	Significance
Body weight	0.00301	0.007537	Significant
Withers height	0.0210	0.026269	Significant
Body length	0.00525	0.008757	Significant
Chest circumference	0.000497	0.002484	Significant
Cannon circumference	0.0869	0.0869	Not significant

Note: The five haplotype–trait association tests were adjusted separately using the Benjamini–Hochberg procedure. An FDR-adjusted *p* value < 0.05 was considered statistically significant.
